# IL-21和Tfh细胞介导NSCLC免疫治疗的研究进展

**DOI:** 10.3779/j.issn.1009-3419.2024.101.19

**Published:** 2024-07-20

**Authors:** Xingkai LIU, Yifan ZHANG, Xin ZHANG, Gonghao HE, Wenke CAI

**Affiliations:** ^1^650032 昆明，中国人民解放军联勤保障部队第九二〇医院胸心外科; ^1^Department of Cardiothoracic Surgery, 920^th^ Hospital of Joint Logistic Support Force of the People's Liberation Army, Kunming 650032, China; ^2^650500 昆明，昆明医科大学; ^2^Kunming Medical University, Kunming 650500, China; ^3^650032 昆明，中国人民解放军联勤保障部队第九二〇医院呼吸与危重症医学科; ^3^Department of Respiratory and Critical Care Medicine, 920^th^ Hospital of Joint Logistic Support Force of the People's Liberation Army, Kunming 650032, China; ^4^650032 昆明，中国人民解放军联勤保障部队第九二〇医院临床药学科; ^4^Department of Clinical Pharmacy, 920^th^ Hospital of Joint Logistic Support Force of the People's Liberation Army, Kunming 650032, China

**Keywords:** 肺肿瘤, 滤泡辅助性T细胞, 白细胞介素21, 免疫检查点蛋白, Lung neoplasms, Follicular helper T cells, Interleukin-21, Immune checkpoint proteins

## Abstract

非小细胞肺癌（non-small cell lung cancer, NSCLC）作为全球范围内频发且极具侵袭性的癌症之一，传统的手术治疗、放化疗及靶向治疗等方法往往由于其固有的局限性，难以遏制病情的进展，导致预后效果并不理想。尽管近年来免疫治疗药物的出现为NSCLC治疗带来了新希望，显示出一定的治疗效果，但当前的疗效仍显不足，无法满足所有患者的治疗需求。因此积极探索新的免疫治疗手段来进一步降低患者的死亡率，已成为NSCLC研究领域的重要方向。本文旨在通过梳理和分析国内外相关文献，系统综述白细胞介素21（interleukin-21, IL-21）和滤泡辅助性T细胞（follicular helper T cells, Tfh）在NSCLC免疫治疗中的抗肿瘤作用，并探讨通过免疫治疗手段调控免疫检查点，以改善NSCLC治疗前景的可能性。

## Abstract (en)

Non-small cell lung cancer (NSCLC) is a prevalent and aggressive global malignancy. Conventional surgical treatments, radiotherapy, chemotherapy, and targeted therapies often fall short in halting disease progression due to inherent limitations, resulting in suboptimal prognosis. Despite the advent of immunotherapy drugs offering new hope for NSCLC treatment, current efficacy remains insufficient to meet all patient needs. Therefore, actively exploring novel immunotherapeutic approaches to further reduce mortality rates in NSCLC patients has become a crucial focus of NSCLC research. This article aims to systematically review the anti-tumor effects of interleukin-21 and follicular helper T cells in NSCLC immunotherapy by summarizing and analyzing relevant literatures from both domestic and international sources, as well as exploring the potential for enhancing NSCLC treatment prospects through immune checkpoint regulation via immunotherapeutic means.

根据世界卫生组织（Word Health Organization, WHO）的数据，肺癌的死亡率在各类癌症中依然高居不下^[1]^。2020年，肺癌死亡人数高达180万，其中非小细胞肺癌（non-small cell lung cancer, NSCLC）占据了主要地位，占80%-85%^[2]^。NSCLC对单克隆抗体免疫检查点抑制剂高度敏感。免疫检查点抑制剂显著改善了一部分局部晚期和转移性NSCLC患者（约20%）的临床结果，并显示出在早期可切除疾病中的新辅助治疗前景^[3]^。然而，并非所有患者都可从中受益，部分患者对免疫治疗的耐药性成为了一大难点^[4,5]^。因此，我们致力于探寻是否存在新的免疫介质能够作用于免疫检查点，从而为NSCLC患者开辟全新的免疫治疗途径。

白细胞介素21（interleukin-21, IL-21）和滤泡辅助性T细胞（follicular helper T cells, Tfh）是NSCLC免疫治疗中的重要参与者。图1展示了Tfh细胞和IL-21参与的抗NSCLC免疫应答。Tfh细胞作为CD4^+^ T细胞的重要亚群，主要栖身于淋巴组织中，其功能受损与NSCLC的发生和发展紧密相关^[6,7]^。IL-21主要由Tfh细胞分泌，能够显著促进CD8^+^ T细胞生成抗肿瘤因子，进而有效攻击和消灭肿瘤细胞^[8]^。并且IL-21还能够增强免疫检查点抑制剂的治疗效果，显著加强免疫细胞的抗肿瘤效能^[9]^。因此，进一步深入研究IL-21和Tfh细胞与免疫检查点之间的联合治疗方案，对于改善NSCLC患者的预后具有极其重要的意义。

**图1:**
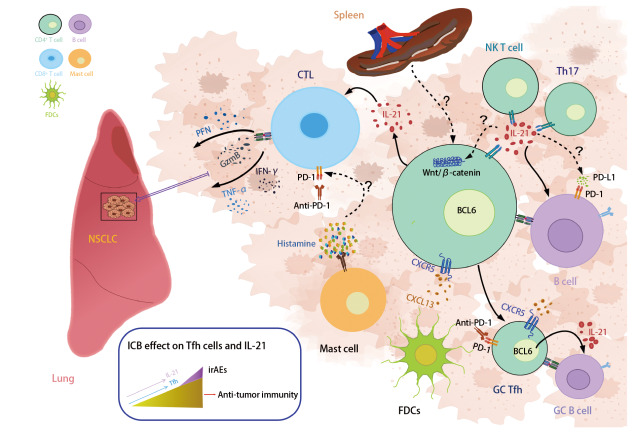
Tfh细胞和IL-21参与的抗NSCLC免疫应答。本图阐释了Tfh细胞和IL-21如何在ICB治疗的影响下促进抗肿瘤免疫应答。实线箭头为已知，虚线问号箭头为未知。如图所示：脾脏中活化的CD4^+^ T细胞分化为早期Tfh细胞，刺激B细胞相互作用并启动早期滤泡外抗体反应。早期Tfh细胞产生IL-21，作用于B细胞增强免疫应答。IL-21还支持CTL细胞功能，促进NSCLC中杀伤肿瘤细胞的物质释放。Tfh细胞调节抗原特异性B细胞进入GC，并使FDCs上的CXCL13与Tfh上的CXCR5相互作用。ICB治疗如抗PD-1可增强Tfh细胞和IL-21功能，但也可能增加irAEs的发生风险。抗肿瘤活性与irAEs之间的关系尚不清楚，但有证据提示两者呈正相关。

## 1 Tfh细胞和IL-21

### 1.1 Tfh细胞的基本特征和功能

Tfh细胞作为CD4辅助性T细胞的一个独特子集，在B细胞的形成和维持生发中心（germinal center, GC）中发挥着核心的作用。这些Tfh细胞通过产生高亲和力的类转换抗体、长寿命浆细胞和记忆B细胞，为免疫系统的有效运作提供了关键支持^[10⇓⇓-13]^。在GC内，Tfh细胞发挥着至关重要的作用。它们通过高度表达趋化因子受体如C-X-C基序趋化因子受体5（C-X-C motif chemokine receptor 5, CXCR5），以及转录因子如B细胞淋巴瘤6（B-cell lymphoma 6, BCL6），精确地定位并调控免疫应答。当CXCR5表达增加时可以促使激活的T细胞迁移到B细胞滤泡中，从而促进Tfh细胞的分化^[14]^。而BCL6的功能不仅能加强Tfh细胞的分化，还能抑制其向其他T细胞亚群的分化^[15]^。同时，Tfh细胞还利用表面上的共刺激受体，例如诱导性协同共刺激分子（inducible synergistic co-stimulation molecules, ICOS）与共抑制受体如程序性死亡受体1（programmed cell death protein 1, PD-1）以进行精细的交互。通过这些复杂的分子相互作用，Tfh细胞能够有效地分泌IL-21等关键细胞因子，从而在免疫应答中执行其多元化的重要功能^[14,16⇓-18]^。因此，Tfh细胞的功能十分重要，对Tfh细胞及其调控机制的深入研究不仅有助于我们深入地理解NSCLC等疾病的发病机制，更有望为开发新型的免疫治疗方法提供重要的科学依据。

### 1.2 IL-21的功能以及在免疫调节中的作用

IL-21是一种结构独特、功能丰富的细胞因子，其核心结构由4个α螺旋束构成，主要源于自然杀伤T细胞、Tfh细胞以及辅助性T细胞17。尽管其他淋巴造血细胞也能产生IL-21，但分泌量相对较低^[10,19,20]^。在活化的T细胞中，尽管BCL6等位基因的消融对T细胞的早期活化和滤泡定位过程影响较小，但其过表达能显著促进IL-21受体的表达，并且BCL6对GC的形成以及Tfh的维持也产生了显著的抑制作用^[21⇓-23]^。此外，在IL-21的作用下，BCL6 mRNA和CXCR5 mRNA的表达均会得到显著上调^[16]^。并且CD4^+ ^T细胞产生的IL-21在慢性病毒感染期间对维持CD8^+ ^T细胞反应具有至关重要的作用。特别是Tfh细胞衍生的IL-21对于维持CD8^+ ^T细胞反应和病毒控制具有不可或缺的作用^[24]^。因此，增强IL-21和Tfh细胞的活性，能够显著提高CD8^+^ T细胞介导的免疫能力，这不仅为疾病的防治提供了新的视角，更为我们抗击各类疾病提供了更全面的治疗策略。

## 2 Tfh细胞和IL-21在肿瘤中的作用

### 2.1 Tfh细胞在肿瘤免疫中的作用

尽管癌症和免疫肿瘤学的研究长期以来主要聚焦于效应CD4^+ ^T和CD8^+ ^T细胞，但近年来，Tfh细胞在多种癌症类型的研究中脱颖而出，成为备受瞩目的免疫细胞群体^[25]^。特别是在NSCLC中，Tfh细胞扮演着举足轻重的角色，它们对B细胞的激活、GC的形成以及抗体的产生具有不可或缺的作用^[17,18,26]^。GC是B细胞成熟的主要场所，Tfh细胞主要功能是调控GC的发育。它通过精细调节GC的大小，限制亲和力低的B细胞进入GC，从而使高亲和力的B细胞在GC内占据主导地位^[14]^。BCL6在Tfh细胞的分化和功能中扮演着举足轻重的核心调控角色，BCL6不仅在Tfh细胞的分化中起作用，而且对于Tfh细胞所支持的GC形成也是必不可少的^[22,25]^。由BCL6诱导产生的CXCR5是Tfh细胞的标志性特征之一，随着CXCR5表达的上调，BCL6表达也会逐渐上调，并且高表达的BCL6能与多种转录因子共同作用来稳定Tfh细胞^[13,16]^。在NSCLC中，Tfh细胞在肿瘤早期阶段常展现出较强的功能活性，然而随着肿瘤的不断恶化，功能性Tfh细胞的数量会显著减少^[27]^。因此，调控Tfh在肿瘤中的数量及活性就显得尤为重要。此外，Tfh细胞和耗竭的CD8^+ ^T细胞的积累情况已被证实可以作为预测多种癌症类型预后的指标^[28,29]^。由此看来，通过对Tfh细胞和CD8^+ ^T细胞的积累与耗竭状态进行监测，我们可以对NSCLC的预后做出前瞻性的预测，从而为患者提供更加精准和有效的治疗方案。

### 2.2 IL-21在NSCLC中的作用

在NSCLC患者的体内，Tfh细胞水平显著上升，并伴随IL-21的产生，然而在肿瘤晚期阶段，Tfh细胞在产生IL-21的功能上可能存在缺陷，其水平的增加反而会导致IL-21减少，这可能会对其免疫应答的效果产生不良影响^[30,31]^。因此挽救Tfh细胞在NSCLC中的功能来提升IL-21的抗肿瘤作用是十分重要的。IL-21作为一种多功能的细胞因子，在增强T细胞和自然杀伤细胞的抗原反应及功能上展现出显著的效果。它不仅能独立刺激IFN-γ的产生，还能与其他细胞因子协同作用，共同增强免疫应答^[32]^。并且，IL-21不仅能通过上调CD4^+ ^T细胞中IL-21的表达，间接促进B细胞产生抗体来参与体液免疫的调节，而且还能够增强CD8^+ ^T细胞的效应功能，从而抑制肿瘤的生长^[33]^。但值得注意的是，并非所有肿瘤新抗原都能触发B细胞-Tfh细胞-IL-21这一通路，因为新抗原必须同时被肿瘤特异性B细胞和CD4^+ ^T细胞所识别才能发挥作用，这一发现揭示了肿瘤新抗原在指导CD4^+ ^T细胞分化方面的新机制^[8]^。体外实验^[33]^表明，CD4^+ ^T细胞产生的IL-21是IL-6促进B细胞抗体产生所必需的。IL-6通过增加IL-21的产生，增强了CD4^+ ^T细胞对B细胞的辅助作用，进而促进抗体的产生，从而抑制肿瘤的进展。此外，IL-21在Tfh细胞的分化过程中也发挥着关键作用，它直接调控B细胞的增殖和类别转换。IL-21R的缺乏会导致抗体反应缺陷和GC形成受损，这进一步强调了IL-21在体液免疫中的重要地位^[34]^。Tfh细胞以CD8依赖性方式发挥抗肿瘤免疫作用，它们产生的IL-21对于维持耗竭T细胞的增殖、活力、细胞因子产生和细胞毒性功能至关重要^[28]^。因此，Tfh细胞的功能活性及其产生的IL-21在NSCLC患者的免疫应答中发挥着复杂而关键的作用。

## 3 免疫检查点

### 3.1 免疫药物在免疫治疗中的应用

多年来，NSCLC的治疗方式十分局限，但免疫治疗的崛起迅速改变了这一治疗格局。表1列出了免疫治疗相关药物的研究进展，清晰地揭示了这些药物在NSCLC治疗中的关键作用以及存在的挑战。其中，PD-1作为NSCLC免疫治疗中的关键生物标志物，其重要性不言而喻^[4]^。PD-1的对应受体细胞程序性死亡配体1（programmed cell death ligand 1, PD-L1）在肿瘤和巨噬细胞的基质区表达丰富，它通过诱导T细胞凋亡和功能衰竭等机制，显著抑制了肿瘤免疫应答^[3]^。PD-1抑制剂主要包括帕博利珠单抗、纳武利尤单抗和阿替利珠单抗等，在PD-L1高表达的NSCLC患者中显示出了显著的治疗效果，极大改善了患者的生存质量，但也存在着一定局限性^[3,5,46,47]^。尽管联用靶向细胞毒性T淋巴细胞相关抗原4（cytotoxic T lymphocyte-associated antigen-4, CTLA-4）抑制剂可能进一步提升疗效，但毒性反应也随之加剧^[3]^。新兴免疫调节剂嵌合抗原受体T细胞免疫疗法（chimeric antigen receptor T-cell immunotherapy, CAR-T）和恶性黑色素瘤相关性抗原A3（melanoma antigen family A3, MAGE-A3）疫苗在NSCLC治疗中也备受关注。然而这些疗法同样面临诸多挑战，如MAGE-A3疫苗在手术切除的NSCLC患者中并未展现出更优的预后效果^[44,45]^。此外，对于IL-2、IL-15等细胞因子的长期疗效和安全性仍需进一步验证。总体而言，免疫治疗为NSCLC患者带来了新的治疗希望，但同时也伴随着治疗对象选择、耐受性以及新兴疗法实施等多重挑战。因此，深入探索免疫治疗的新方向对于改善NSCLC的预后治疗至关重要。免疫介质Tfh和IL-21与免疫检查点的联合应用，或许能为免疫治疗开辟新的道路，为NSCLC的免疫治疗前景带来新的曙光。

**表1:** 免疫治疗药物在NSCLC中的应用

Drug classification	Drug name	Mechanism of action	Adaptation disease	Clinical progression	Approved status	Current problems
Immune checkpoint inhibitors	Nivolumab^[3⇓-5]^	Inhibition of PD-1, blockade of the PD-1/PD-L1 pathway	Non-squamous NSCLC without driver gene mutations and squamous NSCLC	"Nivolumab plus Ipilimumab" (limited PD-L1≥1%) is a grade III recommendation for late first-line treatment, and phase III clinical trials have shown significant improvements in OS and PFS	Approved by the FDA and EMA for second-line treatment of NSCLC, First-line regimens combined with Ipilimumab have also been approved	Drug resistance exists, Combination chemotherapy is not effective, Combining Ipilimumab may result in immune-related adverse events^[37]^
	Pembrolizumab^[3⇓-5]^	Inhibition of PD-1, blockade of the PD-1/PD-L1 pathway	Non-squamous NSCLC without driver gene mutations and squamous NSCLC	"Neoadjuvant Pembrolizumab plus platinum-based chemotherapy plus adjuvant therapy" is a grade II recommendation for perioperative treatment	Approved by the FDA and EMA for NSCLC first and second-line treatment	Primary drug resistance, Patients with low PD-L1 expression had limited efficacy, Easy to cause pneumonia, diarrhea and other adverse events^[45]^
	Atezolizumab^[3⇓-5]^	Inhibition of PD-1, blockade of the PD-1/PD-L1 pathway	Non-squamous NSCLC without driver gene mutations and squamous NSCLC	"Atezolizumab+Bevacizumab+ Carboplatin+paclitaxel" is used as first-and second-line treatment for patients with metastatic NSCLC, phase III clinical trial show obviously improved OS and PFS	Approved by the FDA and EMA for second-line treatment of NSCLC	Patients with high PD-L1 expression had limited efficacy, pneumonia hepatitis colitis immune events^[38]^
	Ipilimumab^[3⇓-5]^	Inhibition of CTLA-4 enhanced T cell activation	Non-squamous NSCLC without driver gene mutations and squamous NSCLC	"Nivolumab plus Ipilimumab" (limited to PD-L1≥1%) is a grade III recommendation for late first-line therapy	Approved by the FDA and EMA. Combined with Nivolumab for the first-line treatment of NSCLC	Limited therapeutic effectiveness, diarrhea and musculoskeletal pain, Lack of predictive biomarkers^[37]^
	Toripalimab^[35]^	Combination of T cell surface molecules, PD-1 and remove PD-1 pathway inhibition of T cells	Non-squamous NSCLC without driver gene mutations and squamous NSCLC	Neoadjuvant and adjuvant therapy with Toripalimab plus Platinum-based chemotherapy is grade I recommendation for perioperative treatment	FDA or EMA not approved, NMPA approved as a first-line treatment for metastatic non-squamous NSCLC combined with chemotherapy	Immune-related adverse events was high
	Tislelizumab^[36]^	Blocking the binding of PD-1 and PD-L1	Non-squamous NSCLC without driver gene mutations and squamous NSCLC	"Tislelizumab+Platinum-based chemotherapy neoadjuvant therapy+adjuvant therapy" is a grade II recommendation for perioperative treatment	FDA approves a combination therapy for the first-line treatment of ES-SCLC; NMPA approves for the treatment of advanced NSCLC	Hypothyroid-related adverse events
	Camrelizumab^[39]^	Blocking the binding of PD-1 and PD-L1	Non-squamous NSCLC without driver gene mutations and squamous NSCLC	"Neoadjuvant Camrelizumab+Platinum-based chemotherapy + adjuvant therapy" is a grade II recommendation for perioperative treatment	Approved by the FDA as neoadjuvant therapy for patients with stage IB-IIIA NSCLC	White-cell count and the neutrophil count was low
	Durvalumab^[40]^	Blocking the binding of PD-1 and PD-L1	Non-squamous NSCLC without driver gene mutations and squamous NSCLC	"Neoadjuvant plus adjuvant therapy with Durvalumab plus Platinum-based chemotherapy" is a grade III recommendation for perioperative treatment	Approved by the FDA and EMA for the treatment of unresectable stage III NSCLC	Skin rash, pneumonia and other adverse events
	Serplulimab^[41]^	Competitive inhibition with PD-1	Squamous NSCLC	"Serplulimab+Albumin paclitaxel+Carboplatin" is a grade I recommendation for late first-line therapy	NMPA approval for squamous NSCLC	Hypothyroidism, rash and immune-mediated lung disease
	Sintilimab^[42]^	Blocking the binding of PD-1 and PD-L1	Drive gene mutation positive NSCLC	"Sintilimab+Bevacizumab+Platinum-based chemotherapy" is a grade I recommendation for late second-line therapy	FDA or EMA not approved, NMPA is approved for the treatment of EGFR mutation-positive locally advanced or metastatic non-squamous NSCLC after EGFR-TKIs treatment failure	Anemia, pneumonia and other adverse events
Immune modulators	MAGE-A3 vaccine^[43]^	Inducing the body to produce specific antitumor immune response	Being evaluated for treatment in patients with NSCLC	Phase III clinical trials	Not approved	Effect of differences, Risk of infection, vascular disease and cancer^[43]^
	CAR-T cell therapy^[44]^	In vitro and activate the immune cells and enhance immune function	Being evaluated in phase I/II clinical trials for NSCLC	The phase I/II clinical trial is ongoing	Not approved	Efficacy and safety of insufficient data, Potential toxicity of nerve toxicity, non tumor targeting and cytokine release syndrome^[44]^
	IL-2, IL-15 cytokines, etc^[4]^	Promote the immune cell activation and function	Being evaluated for treatment in patients with NSCLC	The clinical trials are ongoing	Not approved	Curative effect have limitations, optimal dose is uncertain

MAGE-A3: melanoma antigen family A, 3; CAR-T: chimeric antigen receptor T-cell immunotherapy; OS: overall survival; PFS: progression-free survival; FDA: Food and Drug Administration; EMA: European Medicines Agency; NMPA: National Medical Products Administration; ES-SCLC: extensive-stage small cell lung cancer; EGFR: epidermal growth factor receptor; EGFR-TKIs: epidermal growth factor receptor-tyrosine kinase inhibitors; IL-2: Interleukin-2; IL-15: Interleukin-15.

### 3.2 Tfh细胞与免疫检查点在NSCLC中的作用

PD-1抑制剂已广泛应用于NSCLC及其他恶性肿瘤的治疗，旨在激活并恢复耗竭的T细胞反应。CXCR5诱导的Tfh细胞，以其组成型高PD-1表达为特点，不仅与三级淋巴结构（tertiary lymphoid structures, TLS）的形成紧密相连，更在抗肿瘤免疫应答中起到了至关重要的作用。这些特殊的PD-1 CXCR5 CD4^+ ^T细胞，不仅能够促进CD8^+ ^T细胞和B细胞产生有效的炎症反应，而且能通过抑制PD-1进行精准调控^[43]^。研究^[48]^显示，NSCLC患者中CXCR5 CD4^+ ^T细胞上PD-1的表达水平均显著超出健康个体。尽管活化的T细胞可能短暂地表达CXCR5，但Tfh细胞似乎具有更为稳定的趋化因子受体表达，从而调节体液免疫。活化Tfh细胞的滤泡定位主要依赖于趋化因子受体CXCR5，它能够精准感知由滤泡衍生的ICOS的信号。当B细胞上的ICOS配体表达时，CXCR5能够促进Tfh细胞的运动，确保它们在适当的位置发挥作用^[28]^。此外，PD-1进一步限制Tfh细胞上CXCR3的上调，确保这些细胞主要集中在GC区域，从而优化B细胞间的竞争和亲和力^[49]^。免疫检查点ICOS在Tfh细胞的分化和迁移过程中同样发挥着关键作用。ICOS基因突变与Tfh细胞密切相关，ICOS突变将导致Tfh细胞缺乏，从而影响B细胞分化和体液免疫应答。Tfh 细胞表达的分子PD-1能够使抗原依赖性T细胞-B细胞具有相互作用的稳定性，这有利于与GC的B细胞发生强同源相互作用^[21]^。在肿瘤免疫微环境中，RNA结合蛋白Roquin具有抑制ICOS的能力，Roquin1和Roquin2的联合缺失会导致自发的Tfh细胞和GC的发育^[14]^。值得注意的是，在体内树突细胞中，ICOS会参与BCL6的诱导和Tfh细胞的分化^[16]^。而BCL6对PD-1也具有明确的调控^[25]^。PD-1和ICOS作为Tfh细胞上表达的关键功能分子，它们的表达促使了B细胞的分化和抗体的产生。在此过程中，活化Tfh细胞也起了关键作用^[18,27]^。因此，这些分子与Tfh细胞以及免疫检查点之间的作用对NSCLC的治疗策略有着显著的影响。

### 3.3 IL-21与免疫检查点在NSCLC中的作用

ICOS信号可以促进IL-21的产生，而IL-21则进一步提升了BCL6 mRNA的表达水平，从而在Tfh细胞的发育过程中起到了至关重要的作用^[16]^。特别是在诱导包括PD-1在内的信号分子上调方面，IL-21扮演了关键角色，促进了Tfh细胞的成熟。此外，IL-21还在B细胞以及CD4^+ ^T和CD8^+ ^T淋巴细胞的分化和增殖中起到了不可或缺的作用，展现出强大的抗肿瘤潜力^[50]^。研究^[9]^揭示，IL-21/IL-21R的信号转导可能是通过抑制Wnt/β-catenin信号转导和PD-L1的表达，有效地抑制NSCLC中的细胞增殖、侵袭和迁移，为肿瘤治疗提供了新的策略。值得注意的是，PD-L1的过表达与肿瘤免疫紧密相关，抑制PD-L1能够显著增强抗肿瘤免疫的能力，它通过阻止肿瘤细胞逃避宿主免疫应答来提高治疗效果。此外，还有研究^[9]^表明，用IL-21体外处理NSCLC细胞后，PD-L1的表达水平呈剂量依赖性下调，进一步证明了IL-21能够抑制PD-L1的表达，从而发挥抗肿瘤作用。PD-1与其配体PD-L1之间的相互作用是肿瘤中免疫反应受到抑制的关键因素。目前PD-1/PD-L1检查点抑制剂在NSCLC中已展现出令人瞩目的抗肿瘤活性，为肿瘤患者带来了新的治疗希望^[43]^。但IL-21影响免疫检查点的相关免疫介质仍不完全清晰。我们需要进一步的研究来揭示IL-21在抗肿瘤免疫中的作用机制，从而为肿瘤治疗提供更为精准和有效的策略。

### 3.4 免疫介质作用于免疫检查点对NSCLC的影响

免疫介质在免疫检查点中发挥着重要作用，特别是抗组胺药物在联合接受抗PD-1或抗PD-L1单克隆抗体免疫治疗的肺癌患者中展现出了显著提升生存率的效果^[51]^。多项研究^[52,53]^揭示，肿瘤细胞中组胺及其受体，尤其是组胺受体H1（histamine receptor H1, HRH1）至HRH4呈现高表达状态，这提示它们参与并调控了肿瘤的发生与发展。尤其是HRH4已被证实对NSCLC的上皮间充质转化过程具有显著的抑制作用。研究^[51]^表明，组胺与肿瘤相关巨噬细胞上的HRH1结合会抑制CD8^+ ^T细胞功能，加速肿瘤生长。但组胺H1拮抗剂能中和这种免疫抑制效应，增强抗肿瘤免疫力，提高免疫治疗效果。并且组胺和HRH1的表达在肿瘤微环境中显著增加，并诱导T细胞功能障碍。敲除HRH1或抗组胺治疗可恢复巨噬细胞免疫活性，使T细胞恢复细胞毒性功能，并重新激活免疫治疗反应。巨噬细胞HRH1通过释放Ca^2+^影响T细胞的抑制进而改变抑制性受体T细胞激活抑制物免疫球蛋白可变区结构域（V-domain immunoglobulin suppressor of T-cell activation, VISTA）在细胞膜上的表达。由此看来，HRH1高表达会促进肿瘤生长效应，这可能会诱导免疫治疗耐药性的产生。此外，对于接受免疫检查点抑制剂治疗的NSCLC患者，其血浆样本在体外试验中展现出了对肿瘤细胞侵袭性的差异化影响。针对这些关键的免疫检查点蛋白，免疫检查点抑制剂疗法已成为晚期NSCLC治疗的关键策略^[54]^。并且将HRH1抑制与免疫检查点阻断治疗结合还能增强肿瘤模型的治疗反应^[55]^。此外，组胺与T细胞功能障碍存在紧密关联，组胺表达的增加能够促进癌症的进展。由此看来，组胺表达水平或可成为癌症预后的生物标志物^[56]^。因此，组胺可以作为癌症治疗的潜在靶点，其受体和免疫治疗的联合使用，可能为癌症治疗提供新的潜力。然而鉴于组胺及受体在癌症生物学中的复杂病理生理作用，在将组胺受体引入临床治疗之前，仍需进一步的研究。

## 4 展望

IL-21和Tfh细胞作为NSCLC中至关重要的细胞因子，对肿瘤免疫抑制靶点具有显著影响。本综述主要聚焦于IL-21和Tfh细胞在NSCLC中的免疫作用，深入剖析了IL-21、Tfh细胞与免疫介质组胺对免疫检查点的影响，并概述了它们在NSCLC中的潜在机制。并且，组胺介质与免疫检查点之间的相互作用，与癌症的发展和转移密切相关。组胺还能调节免疫细胞向肿瘤部位的浸润，发挥关键的免疫调节功能。因此，深入研究IL-21、Tfh细胞和免疫介质对免疫检查点的影响，不仅有助于我们进一步理解NSCLC的免疫治疗策略，还可能为NSCLC的诊断和治疗方法提供新的方向。
